# Enhancing feature selection for ordinal outcomes using resampling-based sparse linear discriminant analysis

**DOI:** 10.1093/bioadv/vbag196

**Published:** 2026-07-13

**Authors:** Yin Liu, Ryan Wang, Dong Si

**Affiliations:** Department of Neurobiology and Anatomy, McGovern Medical School, University of Texas Health Science Center at Houston, Houston, TX 77030, United States; McWilliams School of Biomedical Informatics, University of Texas Health Science Center at Houston, Houston, TX 77030, United States; North Creek High School, Bothell, WA 98012, United States; Department of Computing and Software Systems, University of Washington, Bothell, WA 98011, United States

## Abstract

**Motivation:**

High-dimensional biomedical datasets with ordinal outcomes—such as cancer stages or treatment responses—pose significant challenges for feature selection due to strong predictor correlations and limited sample sizes. Sparse Linear Discriminant Analysis (sLDA) is widely used for simultaneous classification and feature selection. However, concerns about model stability and reproducible feature selection persist, particularly in the presence of pronounced collinearity inherent in biomedical data. Consequently, direct application of sLDA often fails to capture a reproducible set of biologically coordinated markers, resulting in signatures that lack robustness and interpretability.

**Results:**

We propose a resampling-based ensemble sLDA framework that integrates bootstrapping and subsampling to improve the stability of feature selection. By aggregating results across multiple resampled datasets, the method identifies features based on Variable Inclusion Probability (VIP) rather than relying on coefficients from standard sLDA. Compared with standard sLDA, this ensemble strategy reduces sensitivity to data perturbation and improves the stability and reproducibility of selected feature sets. Simulation studies demonstrate that the proposed ensemble framework achieves more accurate and consistent recovery of ground-truth predictors compared with the standard (non-resampled) sLDA. Applications to kidney renal papillary cell carcinoma staging and glioma grading datasets further suggest that this framework can improve predictive performance and identify biologically interpretable and reproducible feature sets, highlighting its potential utility for reliable biomarker discovery in precision medicine.

**Availability and implementation:**

The source code used in the study is available via GitHub at https://github.com/ryan-wng/RE-sLDA

## 1 Introduction

Discriminant analysis is a cornerstone of supervised learning, particularly in high-dimensional settings where the goal is to identify a small subset of informative features that effectively distinguish outcome categories. This is especially pertinent in biomedical research, where outcomes are often ordinal—such as treatment responses (Complete Response, Partial Response, Stable Disease, Progressive Disease), cancer stages (Stage I–IV), or clinical risk scores (Low, Medium, High). A common application involves using gene expression data to predict ordinal drug responses, a growing area of interest in pharmacogenetics. The challenge lies in selecting a minimal set of discriminative features that not only enhance interpretability but also improve classification performance for ordinal outcomes. For example, identifying predictive biomarkers that are up- or down-regulated during disease progression can provide valuable insights. Thus, methods that simultaneously perform feature selection and yield accurate classifiers for ordinal labels are highly desirable.

Sparse Linear Discriminant Analysis (sLDA) offers a natural approach due to its close relationship with least squares regression in the classical *p* < *n* setting (Hastie *et al.* 1994, 1995, Mai *et al.* 2012, Wang *et al.* 2013, Ma and Ahn 2021). Inherent to Fisher’s linear discriminant analysis (Fisher 1936), sLDA aims to identify a reduced set of discriminant coordinate functions that optimize class separation using optimal scoring (OS) (Hastie *et al.* 1994). This formulation enables the integration of regularized regression techniques into classification tasks. For example, Clemmensen *et al.* (2011) employed a L1 or lasso penalty to select relevant features for classification. Cardoso and da Costa (2007) transforms the original ordinal classification problem into a series of binary classification problems through a data augmentation step. It enables us to find a single direction vector ***β*** that best separates all binary cut-offs simultaneously. By construction, sLDA respects the inherent ordering of the labels by penalizing large-scale misclassifications (e.g. predicting stage IV for a stage I patient) more heavily than smaller, adjacent errors. Unlike conventional regression-based approaches that treat outcomes as nominal or rely on multiple binary contrasts, sLDA directly leverages the ordinal structure of the response by identifying a single projection that simultaneously separates ordered classes. This leads to a more interpretable and structurally coherent classification rule, while maintaining competitive predictive performance (Cardoso and da Costa 2007).

However, the transition from a theoretical classification framework to a reliable tool for biomarker discovery reveals significant practical hurdles. While sLDA provides a sparse solution, it presents challenges for robust biomarker identification. Its performance is highly contingent on the selection of the tuning parameter (λ). When standard cross-validation (CV) is employed on a single dataset, the “optimal” λ often fluctuates significantly across different data splits, leading to an inconsistent selection of features that may include redundant or noisy predictors (Huber 1973, Hastie *et al.* 1995, Greenman *et al.* 2002).This instability is further exacerbated in high-dimensional biological datasets where features frequently exhibit strong collinearity. Because sparse estimators like lasso are designed to select only a single representative from a group of highly correlated predictors (Xu *et al*. 2011), sLDA may arbitrarily drop functionally relevant biomarkers that belong to the same biological pathway. Consequently, relying on a single iteration of sparse selection often fails to capture the complete set of coordinated biomarkers, resulting in a signature that lacks the robustness and biological interpretability required for reliable biomarker identification.

To address these challenges, we adopt a resampling-based ensemble framework (Efron 1983, Wu 1986, Efron and Tibshirani 1997, Le Teuff *et al.* 2005, De Bin *et al.* 2016). Rather than relying on a single model fit, this approach involves generating multiple “pseudo-samples” from the original data to repeatedly apply the sLDA framework. By aggregating results across these multiple iterations, we shift the focus from a binary “selected or not” decision to a continuous inclusion probability. This resampling mechanism improves model reliability by “averaging out” the variance inherent in sparse estimators, ensuring that the final biomarker signature consists of variables that consistently demonstrate discriminative power across various data perturbations. Consequently, this framework provides a more robust and biologically interpretable foundation to potentially improve biomarker discovery, identifying candidate signatures that are invariant to specific sample compositions and better reflect the underlying disease mechanisms. In general, resampling-based strategies are categorized into bootstrapping and subsampling based on their sampling mechanics. Bootstrapping involves sampling observations with replacement, while subsampling (or *k*-out-of-*n* sampling) involves drawing subsets of data without replacement. The consistency and accuracy of models derived from these resampling techniques have been examined in prior studies (Bickel *et al.* 2012, De Bin *et al.* 2016, Janitza *et al.* 2016, Wallisch *et al.* 2021), highlighting the distinct characteristics of each method.

In recent years, the field of bioinformatics has been significantly advanced by specialized deep learning architectures designed for complex biological prediction tasks. For example, capsule-network-based models, such as pACP-CapsNet (Akbar *et al.* 2026) and TargetAVP-DeepCaps (Akbar *et al.* 2025a), have demonstrated strong performance in identifying anticancer and antiviral peptides by capturing hierarchical spatial relationships within feature sets. Similarly, DeepAIPs-SFLA utilizes a shuffled frog-leaping algorithm for feature fusion (Akbar *et al.* 2025b), while iSucc-SnCNs (Akbar *et al.* 2025c) leverage Siamese nested convolutional networks to improve the site-specific prediction of protein succinylation. In addition, deep learning has shown effectiveness in medical imaging; for instance, Qureshi *et al*. (2025) applied a transfer-learning approach using ResNet-50 to classify osteoporosis from knee X-ray images, illustrating the potential of deep architectures for clinical diagnosis. While these deep learning models highlight the predictive power of modern computational approaches, they also emphasize the need for robust and interpretable feature selection methods. In this study, we address this need by proposing a resampling-based sLDA framework for enhancing feature selection in ordinal outcome settings. Through simulation studies and real-data analyses of the kidney renal papillary cell carcinoma (KIRP) staging and glioma grading datasets, we demonstrate that our resampling strategy potentially improves the stability and interpretability of selected feature sets, while maintaining competitive predictive performance. Furthermore, we implement an efficient multi-threaded computational framework that automates large numbers of parallel resampling iterations. This optimization improves scalability for high-dimensional biomedical applications.

## 2 Methods

### 2.1 Sparse LDA via optimal scoring

Let *X* $\in R^{n\times p}$ denote the input data matrix with *n* observations and *p* predictors. The response vector is *y* =${\;(y_{1},\ldots y_{n})}^{T}$. Each observation belongs to one of the *K* ordinal classes $C_{k},\;k\in \{1,\;\ldots ,\;K\}\;\mathrm{and}\;C_{k}$inherits the natural ordering where $C_{1}<\ldots <C_{k}\;or\;C_{1}>\ldots >C_{k}.$

Originally introduced by Fisher (1936), LDA seeks linear combinations of predictors that maximize between-class variance relative to within-class variance. The discriminant vectors $\beta_{1},\ldots ,\beta_{k-1}$ that are obtained by solving:


$${\mathrm{maximize}}_{\beta_{k}}\{\beta_{k}^{T}\Sigma_{b}\beta_{k}\}$$

$$\mathrm{subject}\;\mathrm{to}\;\beta_{k}^{T}\Sigma_{w}\beta_{k}=1,\;\beta_{k}^{T}\Sigma_{w}\beta_{l}=0\;\forall l<k.$$

where $\Sigma_{w}$ and $\Sigma_{b}$ are within- and between-class covariance matrices, respectively.

Using the OS (Hastie *et al.* 1995), the classification problem is reformulated as a penalized regression.


$${\mathrm{minimize}}_{\beta_{k},\theta_{k}}\{{\left\|Y\theta_{k}-X\beta_{k}\right\|}^{2}+\lambda {\left\|\beta_{k}\right\|}_{1}\}$$

$$\mathrm{subject}\;\mathrm{to}\;\frac{1}{n}\theta_{k}^{T}Y^{T}Y\theta_{k}=1,\;\theta_{k}^{T}Y^{T}Y\theta_{l}=0\;\forall l<k.$$

Here, $\theta_{k}$is a score vector, λ is tuning parameter. The $l_{1}$ penalty induces sparsity in $\beta_{k}$, enabling feature selection. We solve this non-convex optimization using the accelerated proximal gradient algorithm (Clemmensen *et al.* 2011, Atkins *et al.* 2023). The problem is further cast to a binary classification problem as described in Cardoso and da Costa (2007) to handle the ordinal labels. Therefore, a single discriminant vector comes from the result.

### 2.2 Evaluate the performance of feature selection

The performance of our method for feature selection is evaluated by four metrics: Mean Absolute Error (MAE), Accuracy (ACC), Precision, and Recall. MAE is defined as $\mathrm{MAE}=\sum_{i=1}^{n}\left|y_{i}-\hat{y_{i}}\right|/n$. $y_{i}$ and $\hat{y_{i}}$ are the observed and predicted ordinal labels, respectively. MAE quantifies the average deviation between predictions and true labels, making it particularly informative for ordinal outcomes, as it accounts for the magnitude of prediction errors. Accuracy is defined as ACC $=\sum_{i=1}^{n}\left(y_{i}==\hat{y_{i}}\right)/n$, which measures the proportion of exact matches between predicted and observed labels. While ACC provides a straightforward assessment of classification correctness, it does not reflect the ordinal structure of the data. In this analysis, we primarily use MAE to evaluate model performance due to its sensitivity to ordinal differences and use ACC as a complementary metric to report exact classification accuracy. Compared to MAE and ACC, Precision and Recall were used to evaluate the effectiveness of the feature selection process. Precision measures the proportion of selected features that are truly informative, whereas recall measures the proportion of true signal features that are successfully identified. Together, these metrics assess the method’s ability to balance false positives and false negatives under varying selection thresholds.

### 2.3 Cross-validation for the tuning parameters

The tuning parameter λ plays a critical role in shaping the performance of the resulting classifier. To identify its optimal value, we conduct a grid search aimed at minimizing classification error across a range of candidate values. The dataset is initially split into training and testing sets in a 4:1 ratio. The training set is further partitioned into fitting and validation subsets in a 3:1 ratio, resulting in an overall data split of fitting: validation: test = 3:1:1. Within the training set, the fitting and validation subsets are used to tune λ and select the best-performing model. This model is then refitted on the entire training set and evaluated on the test set. We record both the classification error and the number of selected features to assess model sparsity and predictive accuracy.

### 2.4 Feature selection based on bootstrapping

**Figure 1:**
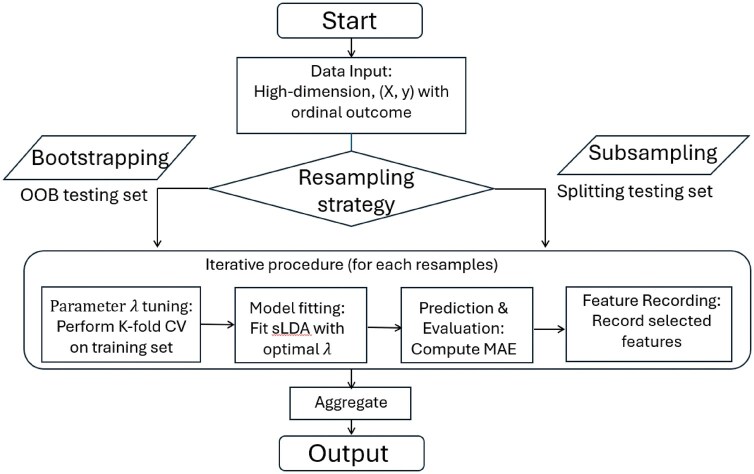
Workflow of sLDA with resampling strategies.

The standard bootstrap, which samples Nobservations with replacement from a population of size N, yields an expected number of unique observations of approximately $N(1-e^{-1})$, or ∼63.2% of the original dataset. The remaining 36.8% are the unique out-of-bag (OOB) observations used for testing (De Bin *et al.* 2016). For consistency with the subsampling-based splitting strategy (Fig. 1), we used 20% of OOB observations as the testing set. This proportion provides a stable assessment of predictive performance while preserving adequate training data, which is particularly important in high-dimensional analyses.

The bootstrapped sLDA procedure consists of the following three steps (Fig. 1):

#### 2.4.1 Step 1: Bootstrap sampling

For each iteration, draw a bootstrap sample of observations from the original dataset with replacement as training set and the rest as testing set based on the testing proportion of the unique OOB observation.

#### 2.4.2 Step 2: Model fitting and lambda tuning

- Perform four-fold CV, using three-folds for training and one-fold for validation, across a predefined grid of λ values to identify the optimal regularization parameter.
- Fit the ordinal sparse LDA model using the optimal λ on the entire training set.

#### 2.4.3 Step 3: Model testing

- Predict class labels for the testing set using the fitted model.
- Record selected features (nonzero coefficients in *β*) and performance metrics (MAE, ACC, Precision and Recall).

Repeat Steps 1–3 for all bootstrap iterations. Aggregate the results by computing the frequency and probability of each feature being selected across iterations, and by summarizing performance metrics. Features are then ranked by their inclusion probability, with rank 1 indicating the most frequently selected—and thus most stable—predictor. The bootstrapped sLDA framework employs resampling and ensemble strategies to address collinearity and stabilize feature selection.

### 2.5 Feature selection based on subsampling

The subsampling approach is similar to bootstrapping, except that it resamples the original dataset *without replacement* during the initial resampling step (Fig. 1). In each resampling iteration, the data was partitioned using a 4:1 split, with 80% of the observation used for model fitting and parameter tuning and the remaining 20% held out for testing. The training portion was used to tune λ and fit the sLDA model, while the held-out test portion was used only for performance evaluation. We then aggregate results across multiple resampling iterations and rank features based on their variable inclusion probabilities.

### 2.6 Computational optimization

Bootstrapping or subsampling of ordinal sLDA model requires extensive computation, primarily due to three factors: (i) a large number of resampling replicates (*B *= 500, or 1000), (ii) a nested K-Fold CV procedure for tuning parameter selection over a grid of candidate values, and (iii) intrinsic complexity of the sparse LDA model. Our framework utilizes specialized iterative optimization techniques—Accelerated Proximal Gradient Descent algorithm (Atkins *et al.* 2023)—where the complexity of a single model fit is approximately *O(k·n·p)*, where *k* represents the number of ordinal classes, *n* is the sample size, and *p* is the number of features. Consequently, the overall computational complexity of the pipeline scales on the order of O(B⋅K⋅k⋅n⋅p). In practice, we further improve efficiency through a multi-threaded implementation, which allows computationally intensive independent operations to be executed in parallel across available CPU cores using the *joblib* Python library. All computations were performed on a Windows system equipped with an Intel Core Ultra 7 processor (16 cores) and 32 GB of RAM. For a representative dataset with *N* = 200, and *P* = 1000 in our real-data analysis, the runtime for a single resampling replicate is ∼0.5 minutes. Consequently, performing 500 resampling iterations would require ∼250 minutes of total computation time under this computing environment.

## 3 Simulation studies

### 3.1 Simulation datasets

To evaluate the performance of our proposed method, we conducted simulation experiments using a framework similar to that of Witten and Tibshirani (2010) and Campbell *et al*. (1991). Let *X* denote an n×p input matrix, with *n* observations and *p* predictors variables. Each observation falls into one of the K ordinal classes, denoted as *C_k_*. For observations in class *C_k_*, data are generated from a multivariate normal distribution, *N* (${µ}_{k},\;\Sigma ),\;$where the class-specific means ${µ}_{k}$ differ across classes. To simulate a co-expression structure, we applied a combination of structured mean differences and covariance-based dependence. Specifically, subsets of informative genes were designed to share similar mean trajectories across ordinal classes, inducing coordinated expression changes associated with disease progression (Simulation Settings 1 and 2). In addition, residual co-expression within each class was introduced through the covariance structure. Specifically, a block-diagonal covariance matrix was imposed, in which the first 10 informative features followed a compound symmetry structure with pairwise correlation ρ, while the remaining features were assumed to be independent with an identity covariance structure. The same covariance structure was used across all classes, with ρ fixed at 0.2 to represent modest but realistic co-expression among informative genes. The vector y contains the corresponding ordinal class labels. We considered 2 simulation settings, described as follows.

#### 3.1.1 Simulation 1: Mixed strong and weak predictors

In this setting, we simulate data of *K* = 3 classes, each containing 20 samples, using the class-specific means ${µ}_{j}\;$proposed by Campbell *et al*. (1991) to induce varying levels of discriminative power. This yields a final dataset with n=60 observations and p=100 predictors. The first 5 features are strong discriminant predictors with large magnitude of ${µ}_{j}\;$difference for ordinal classes, while the next 5 features have a smaller magnitude of ${µ}_{j}\;$difference with less ordinal class discriminant power. Since their class means are ordered according to the class labels, both sets are ordinal features (Han *et al.* 2024), but with varying discriminant power. We set u=1 across the settings.

Features *j* = 1, …,5:


µij= {2u if i ∈C1 u if i ∈C20 if i ∈C3

Features *j* = 6, …,10:


µij={u    if i ∈C1 u/2 if i ∈C20   if i ∈C3

For features *j* > 10: noise features with ${µ}_{ij}∼\;0$ for all classes

#### 3.1.2 Simulation 2: Mixed quasi-ordinal and nominal features

Similarly, for the K=4 class setting, we generated a dataset with n=80 observations and p=100 predictors, including 10 informative features. The first five features follow a partially ordinal (quasi-ordinal) structure, where the class-specific means ${µ}_{j}\;$decrease from class 1 to class 2, while classes 3 and 4 share identical mean values. Features 6–10 are nominal, lacking inherent ordering. In this setup, classes 2 and 3 have the same mean values (higher *μⱼ*), while classes 1 and 4 also share a common but lower mean. Therefore, the first five features are expected to have greater discriminative power than the features 6–10. The combination of two feature groups will provide information to separate the ordinal class.

For features *j* = 1, …,5:


µij= {2u if i ∈C1 u if i ∈C20 if i ∈C30 if i ∈C4

For features *j* = 6, …,10:


µij= {0 if i ∈C1 u if i ∈C2u if i ∈C30 if i ∈C4

For features *j* > 10: noise features with ${µ}_{ij}∼\;0$ for all classes

### 3.2 Simulation results

Table 1 compares the performance of bootstrapping and subsampling sLDA with the standard sLDA (non-resampled) under two simulation settings. Performance was evaluated using predictive metrics (MAE and ACC), feature recovery metrics (Precision and Recall), and the number of selected features [#S($\hat{\beta })$)]. Precision and Recall were computed by tracking the selected features in each resampling iteration and comparing them to the ground truth (Variables 1–10).

**Table 1:** Comparison of bootstrapped sLDA and subsampled sLDA with standard sLDA using simulated datasets.

Simulation setting	Methods	MAE (SE)	ACC (SE)	Precision (SE)	Recall (SE)	#s($\hat{\beta })$ (SE)
1	Bootstrapping	0.168(0.0028)	0.832(0.0029)	0.718 (0.0086)	0.959 (0.0029)	16.8 (0.37)
	Subsampling	0.164(0.0025)	0.836(0.0024)	0.687 (0.0087)	0.960 (0.0028)	17.6 (0.33)
	Standard	0.2 (NA)	0.8 (NA)	0.416 (NA)	0.5 (NA)	12 (NA)
2	Bootstrapping	0.471(0.0044)	0.562(0.0031)	0.401 (0.0077)	0.840 (0.0052)	30.5 (0.70)
	Subsampling	0.465(0.0046)	0.569(0.0032)	0.405 (0.0078)	0.835(0.0064)	30.5 (0.73)
	Standard	0.7 (NA)	0.4 (NA)	0.333 (NA)	0.7 (NA)	21 (NA)

The table reports the mean and standard error (SE) of MAE, ACC, Precision, Recall, and the number of selected features across 1000 resampled datasets. Feature selection is quantified by the number of nonzero coefficients, denoted as #s(*$\hat{\beta })$*).

In both settings, bootstrapping and subsampling achieved comparable predictive accuracy and consistently outperformed the standard sLDA. In Setting 1, both resampling approaches yielded lower MAE and higher ACC relative to the standard method, with subsampling showing a modest advantage. In Setting 2, all methods exhibited higher error rates and lower accuracy than those in Setting 1, reflecting the increased complexity of this scenario, where quasi-ordinal and nominal features jointly define the class structure. Nevertheless, the relative performance pattern remained consistent, with resampling-based methods outperforming standard sLDA. A similar trend was observed for feature selection. Both bootstrapping and subsampling demonstrated substantially improved Precision and Recall compared with standard sLDA, which exhibited both false discoveries and incomplete recovery of the true informative features. This performance gap suggests that standard sLDA may be more sensitive to a single data partition in this setting, whereas resampling-based approaches improve feature selection through aggregation across multiple resampled datasets.

**Figure 2:**
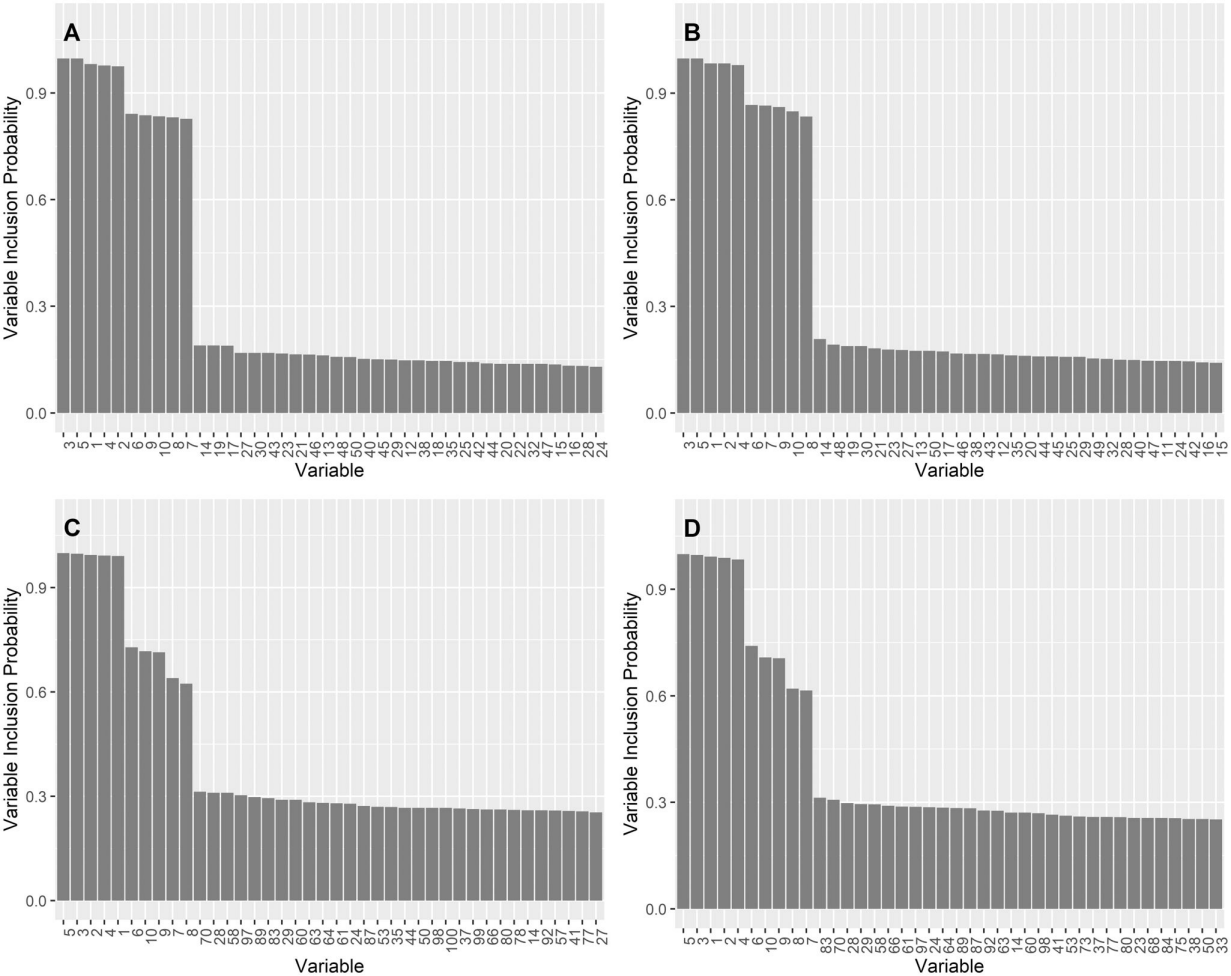
Feature stability measured by VIP in simulation setting 1 (A and B) and 2 (C and D) (showing the top 40 features). (A and C) are from bootstrapping; (B and D) are from subsampling.

To further assess the selection stability, we constructed ensemble-based feature rankings using Variable Inclusion Probabilities (VIP), defined as the proportion of times each predictor is selected across resamples (Fig. 2). Both resampling methods demonstrate a highly consistent rank ordering, with total rank correlation coefficients of 0.95 and 0.89 for Simulation 1 and 2, respectively. The strongest predictors (Variables 1–5) exhibited inclusion probabilities exceeding 0.9 across all experimental conditions. Weaker ordinal predictors and nominal features (Variables 6–10) followed in the ranking hierarchy; notably, quasi-ordinal predictors exhibited higher VIPs than the nominal ones in simulation setting 2, reflecting the framework’s enhanced sensitivity to ordinal data structures.

Furthermore, both resampling strategies effectively suppressed stochastic noise, as inclusion probability dropped precipitously for all features beyond the 10 informative features. By selecting features based on the distinct numerical “gap” or “elbow” in the VIP distribution, the ensemble method effectively filtered out the high-variance noise typically captured in individual runs. Consequently, our method achieved a precision and recall of 1.0 across both simulation settings, ensuring stable and comprehensive feature selection that significantly outperforms standard sLDA.

## 4 Real data analysis results

In addition to simulation studies, we applied bootstrapping and subsampling to two publicly available gene expression datasets to evaluate its performance in real-world biomedical classification tasks. The first dataset, a RNA-seq dataset from TCGA study, contains gene expression profiles of tumor samples from patients with KIRP (Broad Institute 2016). It includes 291 samples categorized into 4 pathological stages (stage 1, stage 2, stage 3, and stage 4). The second dataset, GSE4290 (Sun *et al.* 2006), profiles primary human Glioma samples using the Affymetrix HG-U133 plus 2.0 array. It includes 180 samples categorized into four ordinal grades (“Normal” ≺ “Grade II” ≺ “Grade III”’ ≺ “Grade IV”).

Both datasets contain over 20,000 genes. To reduce computational burden, we applied a high-dimensional screening method (Cui *et al.* 2015) to select the top 1000 genes or probes based on deviations between class conditional and marginal distributions. This model-free screening step was performed once on the full dataset. Although the procedure uses class label information, it does not impose an ordinal structure on the outcome. Rather, it serves as a preliminary dimensionality-reduction step. The ordinal relationships among outcome categories were subsequently captured by sLDA within the resampling framework. We acknowledge that this prescreening strategy may introduce some degree of information leakage because all samples contribute to the definition of the candidate feature set; however, the same screened feature set was subsequently used as input for all compared methods, including standard sLDA without resampling and resampling-based sLDA approaches. Therefore, while the prescreening step may influence absolute performance estimates, any potential advantage introduced by the prescreening step would be shared across all methods and is unlikely to materially affect the relative comparisons among methods. For each dataset, we conducted 500 resampling iterations and genes were then ranked according to their variable inclusion probabilities.

As shown in Table 2, bootstrapped sLDA consistently achieved lower MAE and higher accuracy than both subsampled and standard sLDA across the two datasets. VIP-based gene rankings were highly consistent between the two resampling methods, with rank correlations exceeding 0.91 for both KIRP and Glioma datasets. These findings align with results from our simulated datasets; consequently, the subsequent analysis utilizes gene lists derived from bootstrapping.

**Table 2:** Comparison of bootstrapped sLDA and subsampled sLDA with standard sLDA using real datasets.^a^

Datasets	Methods	MAE	ACC	#s($\hat{\beta })$
KIRP stage	Bootstrapping	0.409 (0.0063)	0.735 (0.0026)	56 (1.0)
	Subsampling	0.473 (0.0029)	0.708 (0.0019)	61 (1.4)
	Standard	0.538 (NA)	0.634 (NA)	49 (NA)
Glioma grade	Bootstrapping	0.425 (0.01)	0.650 (0.03)	102 (4.4)
	Subsampling	0.497 (0.04)	0.596 (0.07)	214 (8.9)
	Standard	0.543 (NA)	0.571 (NA)	56 (NA)

a The table reports the mean and SE of MAE, ACC, and the average number of features with non-zero coefficients selected from 500 resampling iterations, denoted as #s(*$\hat{\beta })$*).

**Figure 3:**
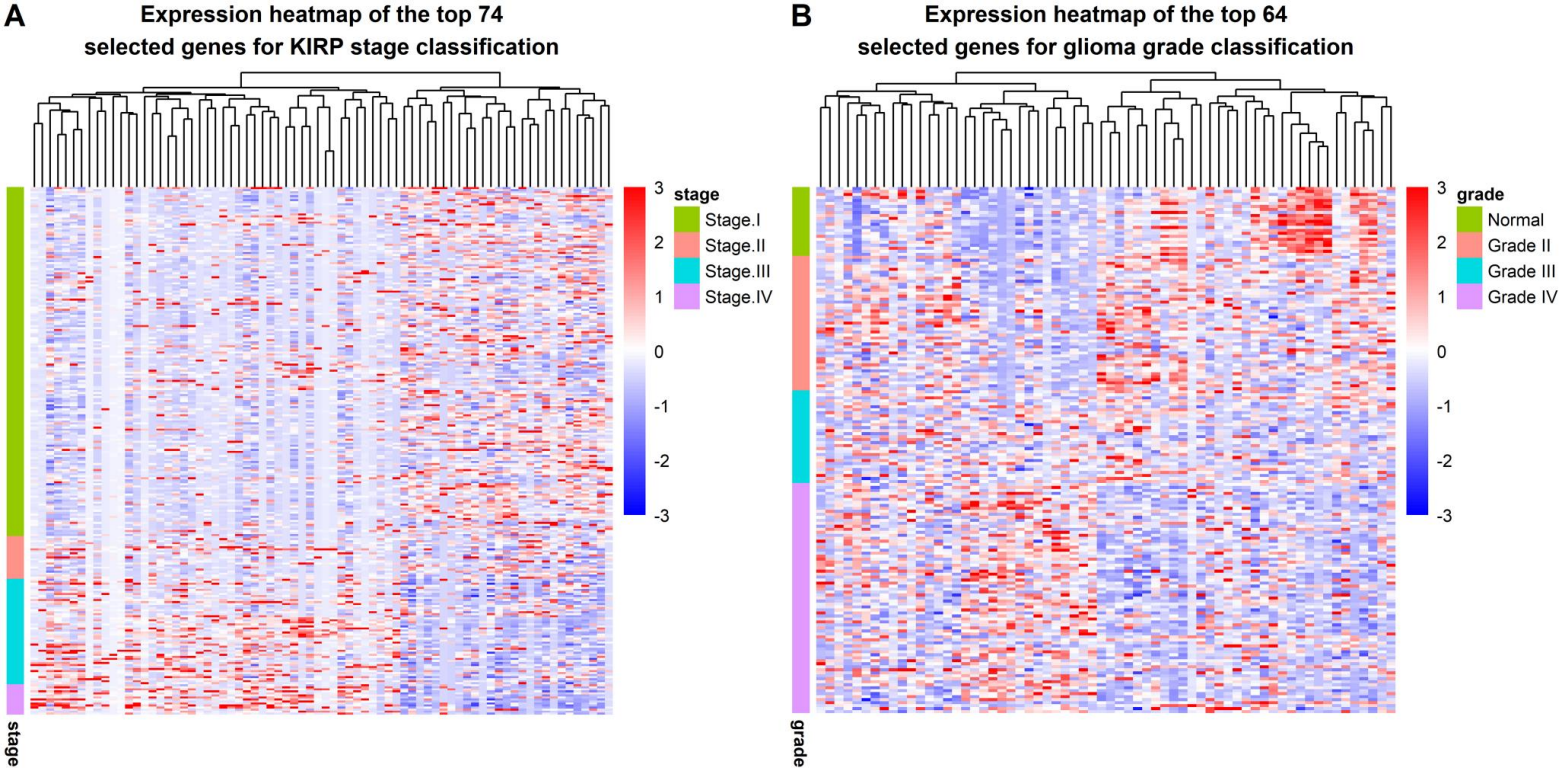
Heatmap of selected genes using bootstrapping sLDA. (A) KIRP dataset (B) Glioma dataset.

**Figure 4:**
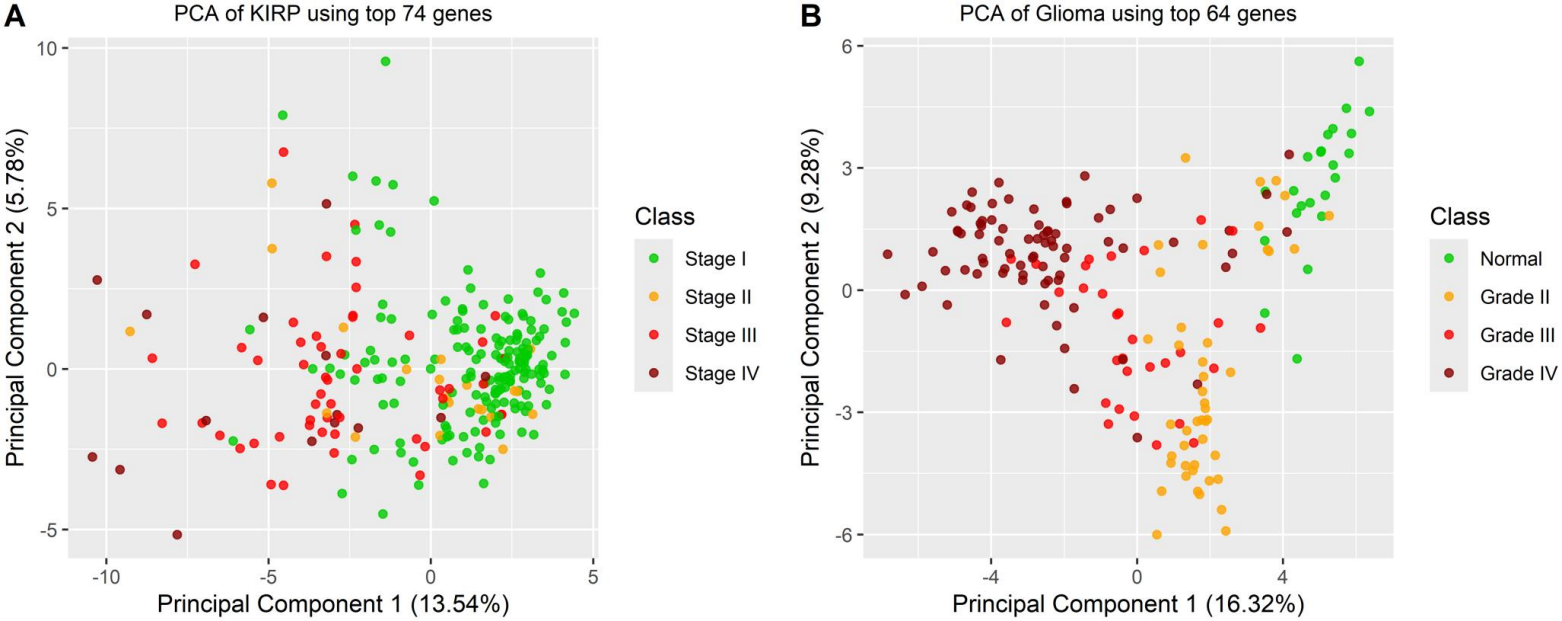
Projection of the datasets onto the 2D subspace spanned by the first two principal components based on the top selected genes via bootstrapping. (A) KIRP dataset (B) Glioma datasst.

We examined the expression patterns of the selected genes using complementary visualization approaches, focusing on the top-ranked gene sets identified by the VIP-based selection procedure. To determine the optimal VIP score cutoff, we applied a derivative-based elbow detection method (Satopaa *et al.* 2011) to identify candidate points at which the VIP curve showed a marked change in slope. For downstream analysis, we selected a gene set size within the range of 50–100 features, balancing model parsimony with the need to retain sufficient biological information for pathway-level analysis. This data-driven approach yielded final selection of 74 genes for the KIRP dataset and 64 genes for the Glioma dataset. The corresponding VIP elbow plots are provided in Fig. S1, available as supplementary data at *Bioinformatics Advances* online, and the selected gene lists are provided in File S1, available as supplementary data at *Bioinformatics Advances* online. Heatmaps and principal component analyses (PCA) were used to assess how these genes are associated with ordinal tumor stage or grade. Results for the top genes with high variable inclusion probabilities are presented in Figs 3 and 4.

Figure 3A displays the expression profiles of the top-ranked genes in the KIRP dataset, with samples ordered by ordinal tumor stage. Genes clustered on the left were predominantly overexpressed in high-stage tumors, whereas those on the right show elevated expression in low-stage samples. Figure 3B presents similar patterns in the Glioma dataset, where gene expression levels are also associated with tumor grade; e.g. the rightmost gene cluster exhibits reduced expression in Grade IV samples. Figure  4 projects the samples onto a low-dimensional subspace using PCA based on the top-ranked genes selected via bootstrapping. The resulting projection reveals clear trajectories corresponding to tumor stage or grade progression. Specifically, in Fig. 4A, lower-stage samples (Stages I–II) cluster toward the right, while higher-stage samples (Stages III–IV) concentrate on the left. In Fig. 4B, normal samples cluster toward the upper left, high-grade tumors (Grade IV) toward the right, and intermediate grades (Grades II–III) occupy an intermediate position.

To validate the biological relevance of the selected genes, we performed Gene Ontology (GO) enrichment analysis (File S2, available as supplementary data at *Bioinformatics Advances* online). The results suggest that the resampling-based approach can identify more biologically coherent feature sets than standard sLDA. Specifically, for the KIRP dataset, the selected genes showed significant enrichment in biological processes related to epithelial morphogenesis, vascular relaxation, and neutrophil differentiation, suggesting potential involvement of tissue remodeling, angiogenesis, and inflammatory signaling. Additionally, tight post-transcriptional control was evident through simultaneous oncogenic transcript stabilization and accelerated poly(A) tail shortening of regulatory mRNAs. For the Glioma dataset, the enriched terms were related to developmental dedifferentiation (e.g. pituitary gland development), synaptic plasticity regulation, and lipid metabolic processes, including chylomicron assembly, which can be relevant to tumor progression and metabolic reprogramming. In contrast, when applying a standard sLDA without resampling, no statistically significant GO terms were identified under the same enrichment analysis criteria. This comparison suggests that the resampling-based approach may improve the interpretability of feature selection and facilitate the recovery of biologically related genes, including potentially correlated genes within the same functional pathway.

## 5 Discussion and conclusion

This study introduces a resampling-sLDA framework designed to address the challenges of feature selection in high-dimensional datasets with ordinal outcomes. Standard sLDA provides an approach for ordinal classification and feature selection, but the selected feature sets may be sensitive to data perturbations, particularly in high-dimensional settings with correlated predictors. To address this limitation, our framework integrates resampling strategy with VIP to improve both the robustness and reproducibility of feature selection.

The transition from theoretical classification to reliable feature selection requires more than high accuracy; it demands stability and interpretability. In biomedical applications, a selected feature signature is only useful if it remains consistent across different patient cohorts. By shifting the focus from a single model fit to a resampling-based consensus, our approach reduces sensitivity to sampling variation and provides a more stable ranking of candidate features. This strategy may be particularly useful in genomic studies, where correlated genes may participate in the same biological pathway. Rather than selecting only one representative feature from a group of correlated ones, the resampling-based framework can help retain multiple functionally related features, thereby improving biological interpretability of the selected feature sets.

Our analysis revealed a critical distinction between simulated and real-world performance. While simulation studies showed comparable results between bootstrapping and subsampling strategies, our analysis of the KIRP and Glioma datasets revealed a performance distinction in these specific contexts. In these two datasets, bootstrapping demonstrated higher predictive accuracy and a more conservative, sparse feature selection compared to subsampling. This observation suggests that bootstrapping may be particularly useful for genomic datasets with complex correlation structures among features. However, because this trend was observed only in these two real datasets, additional studies across a broader range of cancer types and ordinal biomedical outcomes are needed to further evaluate its generalizability.

Several limitations of this study should be acknowledged. First, although our multi-threaded architecture substantially reduces wall-clock time, the computational cost of the ensemble-based sLDA framework remains higher than that of standard sLDA model, which may limit its application in resource-constrained settings. Second, the proposed model assumes that the underlying data structure can be effectively captured by linear discriminants; therefore, in cases that involve highly complex or nonlinear relationships, kernel-based or deep-learning approaches may provide complementary advantages. Third, the initial high-dimensional screening step was performed on the full dataset prior to resampling, which may introduce some degree of information leakage and affect absolute performance estimates. However, because the same prescreened feature set was used for both standard sLDA and resampling-based sLDA approaches, it is unlikely to materially alter the relative comparisons among methods. Thus, although a fully nested screening strategy, in which the high-dimensional feature screening procedure is performed within each training replicate, would provide a stricter assessment of absolute prediction performance, we do not expect it to change the main comparative conclusions under the same preprocessing framework. Fourth, although the proposed framework achieved encouraging performance on the KIRP and glioma datasets, the absence of validation using fully independent external cohorts limits the assessment of generalizability. Finally, the genes identified in this study require further functional biological validation, such as wet-lab experiments, to confirm their potential clinical relevance.

These limitations suggest several directions for future work. First, the ensemble sLDA model could be extended to multi-omics data integration, enabling joint analysis of transcriptomic, genomic, epigenomic, and proteomic features. Second, the proposed stability-focused feature selection strategy could be combined with machine learning or deep learning models to balance predictive performance, interpretability, and reproducibility. Third, a fully nested screening strategy could be incorporated to further minimize potential information leakage and provide a stricter assessment of feature selection robustness. Fourth, validating the proposed framework in larger, multi-center cohorts will be important to further evaluate its robustness and clinical applicability. Finally, continued optimization of the computational pipeline, including improved parallelization and efficient implementation, may further improve scalability for large high-dimensional biomedical datasets.

## Supplementary Material

vbag196_Supplementary_Data

## Data Availability

The data and source code are available via GitHub at https://github.com/ryan-wng/RE-sLDA.
