# Supplementary figures and images for: Enhancing feature selection for ordinal outcomes using resampling-based sparse linear discriminant analysis
Source: Bioinform Adv. 2026 Jul 13;6(1):vbag196. doi: 10.1093/bioadv/vbag196 (PMC13401443; doi:10.1093/bioadv/vbag196)

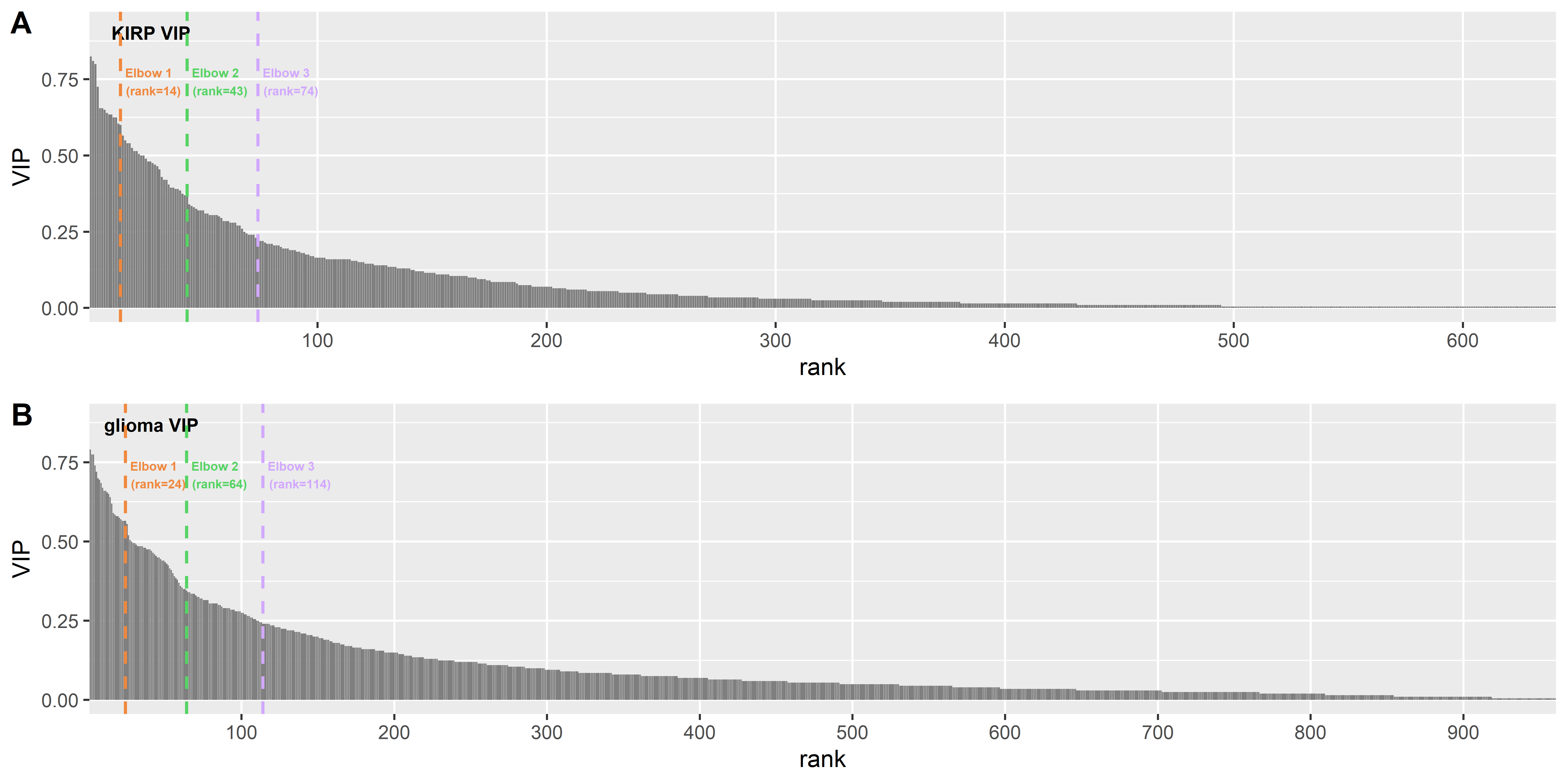

Supplement: vbag196_Supplementary_Data [file vbag196_supplementary_data.zip › SupplementaryFigure_1.tif]
